# Editorial for the Special Issue on Advancing MEMS/MOEMS Sensors: AI-Driven Optimization and Emerging Applications

**DOI:** 10.3390/mi17101157

**Published:** 2026-09-30

**Authors:** Qianbo Lu

**Affiliations:** School of Flexible Electronics (SFE), Northwestern Polytechnical University, Xi’an 710072, China; iamqlu@nwpu.edu.cn

MEMS and MOEMS turn physical and biochemical changes into measurable signals using small chips. In MOEMS, optical cavities make mechanical motion easy to read optically [1], which enables high-resolution chip-scale accelerometers and broadband measurements limited by thermomechanical noise [2,3]. MEMS gravimeters have measured Earth tides [4], wearable sensor arrays have analyzed sweat in multiplexed formats [5], and MEMS scanning mirrors have become key components in LiDAR [6]. These examples point to a common problem: miniaturization and sensitivity matter, but so do bandwidth, noise, power, stability, and manufacturability. A useful sensor must deliver reliable information under all of these constraints.

AI and computational optimization can help address these coupled requirements. Inverse design identifies optical structures that perform a desired function [7], and physics-informed machine learning merges data with physical knowledge for forward and inverse problems [8]. In MEMS, deep reinforcement learning and neural network surrogate models have explored disk resonator gyroscope topologies [9], while neural network-assisted multi-objective optimization has guided resonant accelerometer design [10]. Multistage optimization of free-form anti-springs in a MOEMS gravimeter shows how algorithm-guided design can be integrated with manufacturability and optical readout [11]. Hybrid methods that combine signal decomposition with neural networks have also been used for temperature-drift compensation [12]. These tools work at different stages of sensor development. Their value depends on good physical models, representative data, and validation under real operating conditions.

This Special Issue, “Advancing MEMS/MOEMS Sensors: AI-Driven Optimization and Emerging Applications,” collects eight papers: two reviews and six research articles. They cover AI-enhanced biomedical monitoring, quantum-enabled sensing, high-temperature pressure sensing, electric-field calibration, micro-optical actuation, low-noise readout electronics, and wafer-level integration. Together, they treat intelligence as part of the measurement system, not as a separate algorithm. Data interpretation and computational design are discussed alongside transducers, interfaces, and fabrication.

Cai et al. (Contribution 1) review continuous monitoring with AI-enhanced BioMEMS. They consider sensing performance, sustainable power, and predictive analytics, along with survey-sensing materials, micro/nanoscale architectures, and piezoelectric, triboelectric, and moisture-based energy harvesting. They also discuss how AI can find patterns and anomalies in continuous data. The review is careful about maturity: fully integrated, self-powered BioMEMS with AI analytics are still at an early stage. It offers a roadmap, not a claim that long-term autonomous operation is already solved.

Feng and La (Contribution 2) review machine learning-assisted sensing for COVID-19 monitoring. They introduce relevant learning algorithms, survey their use with electrochemical and optical biosensors, and discuss challenges and future directions. The review focuses on the link between signal acquisition and data interpretation in infectious-disease monitoring. It shows how learning methods can support biosensor development while also stressing the validation needed to ensure dependable performance in healthcare applications.

Rezinkina et al. (Contribution 3) study miniature electromagnetic and mechanical resonators for acceleration measurements using nitrogen-vacancy color centers. Using mathematical and physical modeling, they analyze how resonator configuration and parameters affect the sensing response. The electromagnetic resonators provide microwave excitation of the color centers, and the mechanical resonators support acceleration measurement. The work gives design guidance for combining micromechanical elements with quantum-sensitive readout. It also makes a broader point: the excitation and mechanical subsystems must be developed together, not as separate pieces, when building quantum sensors [13].

Prio et al. (Contribution 4) address pressure measurement in harsh environments with an AlGaN/GaN heterojunction field-effect transistor embedded in a MEMS diaphragm. They combine experiments, finite-element simulations, and theory to study the pressure-dependent electrical response. The sensors show gate bias-dependent sensitivity and repeatable operation over multiple pressure cycles at temperatures up to 300 °C, with a response time below 10 ms. The results show how wide-bandgap materials and transistor-based transduction can push MEMS pressure sensors beyond conventional laboratory conditions.

Ren et al. (Contribution 5) tackle another environmental problem: accurate electric-field measurement when ion flow is present. Using a controlled platform that varies the nominal electric field and ion current independently, they characterize sensitivity changes and the output drift of a MEMS electric-field sensor under DC ionized-field conditions. A joint calibration that includes ion current density reduces the maximum relative error from 29.28% to about 5.07%. The study explains why calibration based only on electrostatic conditions can fail in high-voltage direct-current environments.

Tan et al. (Contribution 6) present a piezoelectric thin-film actuator for dynamic tuning of micro-optical cavities. Their island-circular structure combines a lead zirconate titanate film with metal electrodes, and particle swarm optimization improves displacement while reducing gravity-induced deformation. Simulations and experiments characterize the actuator dynamics, including a first-order natural frequency of about 5.8 kHz. Integration into a Fabry–Pérot microcavity demonstrates active modulation of the optical path length. This work connects computational optimization, fabrication, and optical validation, contributing to the development of controllable MOEMS cavity systems.

Gong et al. (Contribution 7) focus on the electronic interface: a low-noise analog front-end circuit for capacitive MEMS accelerometers. A fully differential architecture with chopper stabilization suppresses low-frequency noise and reduces parasitic coupling. The circuit is fabricated in a 0.18 μm CMOS process and co-packaged with a silicon-on-glass accelerometer for optical image stabilization. The reported sensitivity of 342 mV/g and dynamic range of 88 dB show why the readout circuit and the mechanical sensing element should be designed as one measurement system.

Li et al. (Contribution 8) address fabrication and integration of three-dimensional MEMS structures through wafer-level stacking that combines wet/dry hybrid etching with low-temperature adhesive bonding. Their process includes convex-corner compensation, steps to improve large-area dry-etching uniformity, and controlled alignment and adhesive curing. They validate the approach by fabricating a symmetrically stacked, triple-layer MOEMS accelerometer sensing element, with a reported noise floor of 0.40 μg/√Hz. The study ties process development to measured sensor performance and offers a manufacturing route for complex hollow structures and multilayer microsystems.

What emerges from these papers is that progress in developing MEMS/MOEMS is best judged at the system level. The BioMEMS reviews connect sensing to power and data interpretation. The studies on resonators, pressure sensors, and calibration deal with how transduction behaves under real operating conditions. The papers on actuators, circuits, and stacking show that implementation details often determine whether a good idea becomes a useful device. Computational intelligence helps most when it supports these physical foundations, helping researchers make design trade-offs without hiding the mechanisms or limits of the final device.

The priorities arising from this research are as follows: AI-assisted design should include fabrication tolerances, noise, and environmental variability from the start, not just optimize nominal sensitivity [7,8,11]. Learning-based interpretation and compensation should be tested on independent measurements and under changing operating conditions, with uncertainty and computational cost made explicit [8,12]. Energy-aware architectures, reproducible calibration, and reliable wafer-level integration will remain necessary for sustained operation outside the laboratory. I hope this Special Issue encourages closer collaboration among researchers in micromechanics, photonics, electronics, materials, and AI, and helps develop compact sensing systems whose intelligence is matched with physical reliability.
